# Doctor–patient communication in Southeast Asia: a different culture?

**DOI:** 10.1007/s10459-012-9352-5

**Published:** 2012-02-08

**Authors:** Mora Claramita, Mubarika D. F. Nugraheni, Jan van Dalen, Cees van der Vleuten

**Affiliations:** 1 Medical Education Department, Faculty of Medicine, The Skills Laboratory, Universitas Gadjah Mada (UGM), Grha Wiyata Building 3rd floor, Jalan Farmako Sekip Utara, Yogyakarta, 55281 Indonesia; 2 Faculty of Cultural Sciences, Universitas Gadjah Mada (UGM), Yogyakarta, Indonesia; 3 School of Health Professions Education, Maastricht University (MU), Maastricht, The Netherlands

**Keywords:** Doctor–patient communication, Southeast Asian culture, Intercultural communication

## Abstract

Studies of doctor–patient communication generally advocate a partnership communication style. However, in Southeast Asian settings, we often see a more one-way style with little input from the patient. We investigated factors underlying the use of a one-way consultation style by doctors in a Southeast Asian setting. We conducted a qualitative study based on principles of grounded theory. Twenty residents and specialists and 20 patients of a low or high educational level were interviewed in internal medicine outpatient clinics of an Indonesian teaching hospital and two affiliated hospitals. During 26 weeks we engaged in an iterative interview and coding process to identify emergent factors. Patients were generally dissatisfied with doctors’ communication style. The doctors indicated that they did not deliberately use a one-way style. Communication style appeared to be associated with characteristics of Southeast Asian culture, the health care setting and medical education. Doctor–patient communication appeared to be affected by cultural characteristics which fell into two broad categories representing key features of Southeast Asian culture, “social distance” and “closeness of relationships”, and to characteristics categorized as “specific clinical context”. Consideration of these characteristics could be helpful in promoting the use of a partnership communication style.

## Introduction

Studies in Western settings have shown that open and clear communication between health providers and patients can facilitate optimal delivery of health care **(**Maguire and Piceathly 2002). Mutual understanding between doctors and patients can be enhanced when both parties actively engage in dialogue and exchange of information during consultations (Charles et al. 1999; Makoul and Clayman 2006). This can be characterized as a partnership consultation style. Recent studies in non-Western contexts, including Southeast Asia, revealed that in those settings too this consultation style is generally considered preferable (Kiguli et al. 2011; Claramita et al. 2011a, Moore 2009).

However, in Southeast Asian settings, there are signs of discordance between the preferred communication style and the prevailing style. The latter is best *described* as paternalistic or one-way style, with a dominant role for the doctor (Claramita et al. 2011b). This could reflect the doctor’s sense of superiority to the patient within the context of the consultation or the generally large educational gap between doctors and patients. Barriers in implementing the desired partnership style were revealed by a qualitative study conducted earlier: high patient load, patients’ unpreparedness for a participatory consultation style and doctors’ lack of communication skills (Claramita et al. 2011a). But other cultural factors may also be involved.

Studies of the role of cultural factors in doctor–patient communication usually focus on differences between individual doctors and patients. The wider the cultural divide the less satisfied patients tend to be (Saha et al. 1999; Ramirez 2003; Haviland et al. 2005). However, little is known about the dilemma facing Southeast Asian doctors, many of whom studied at Western universities where they are taught to use a partnership communication style. There are cultural barriers to applying this style in a Southeast Asian context with its strong hierarchical culture. One might question whether the partnership communication style advocated in the Western communication literature can be simply transposed to or even whether it seems the most appropriate style for the Southeast Asian context. This problem can be illustrated by the concept of “Equity”, which is central to a partnership communication style, but which is absent from the accepted norms, values and beliefs of Southeast Asian cultures (Harmseen et al. 2003; Geertz 1976).

Contrary to what one would expect based on cultural factors, however, studies conducted in Southeast Asian settings report that both doctors and patients prefer a more egalitarian partnership communication style, even though it may not quite fit the culture. In order to reconcile this apparent incompatibility of communication ideal and contextual, cultural factors, it is important to understand the main factors that are at play here. Key issues to be investigated are whether and to what extent patients accept and perhaps even prefer the prevailing paternalistic consultation style, whether patients’ educational background plays a role in this and which cultural characteristics are important (Haviland et al. 2005).

In order to develop a more suitable communication skills model tailored to the context of Southeast Asia it is essential to find out key cultural issues related to the prevailing paternalistic communication style. Rather than a Western communication skills guideline, a communication skills model that is suited to interaction between people in another culture can be of more benefit for training medical students and young doctors to communicate more effectively to the patients in their context (von Fragstein et al. 2008).

We designed an interview study based on principles of grounded theory in which we used purposeful sampling and iterative qualitative data analysis to explore the use of the one-way consultation style in Indonesian tertiary health care. We looked for factors that could shed light on the gap between aspiration and reality, and point the way to a solution that fits the East Asian context.

## Background characteristics of Southeast Asian culture

The teaching hospital in which the study was conducted serves the Javanese community on Java Island, Indonesia. For centuries, from the earliest days of the Silk Road (the international trade route between India and China), Java has been a multicultural society comprising 350 ethnic groups speaking 164 different local languages (Elisseeff 2001).

Javanese multiethnic society is probably most aptly characterized as a traditional agrarian culture. Unpredictable harvests in the past have given rise to a culture of numerous ceremonies around the expression of hopes and wishes, and with a marked tolerance of uncertainty (Geertz 1976; Elisseeff 2001; Iragiliati 2006). The future is viewed as unpredictable and there is general avoidance of structured schedules and deliberate planning. Relationships like those between doctor and patient are expected to follow unspoken rules of behaviour in which value is placed on politeness and maintaining a positive etiquette for the present moment rather than on the development of a strong long-term relationship based on openness and mutual trust (Iragiliati 2006; Galanti 2008). In brief, in social relationships short term goals are more salient than the long term perspective. Other core values are strong support from family and community social hierarchy and the respect accorded to those who have lived the longest, i.e. elderly people, also referred to as the elders (Hofstede 2003).

The history of modern medicine in Southeast Asia began in the mid 1800s, when it was introduced by Western colonial rulers (Zeichner 1988). Today, Southeast Asian doctors continue to practise medicine according to Western principles, despite some discordance with Southeast Asian cultural characteristics. This study was conducted as part of a search for ways to reconcile the apparent discrepancies between values of Western medicine and the values of a non-Western culture (Claramita et al. 2011a, b).

### Study setting

Doctor patient communication has mostly been studied in Western family practice contexts. In Indonesian health care, the role of the family doctor as a gatekeeper to health care is unknown and there is no setting that is similar to that of Western family practice (Gan et al. 2004). We therefore looked for the nearest equivalent to this setting and conducted our study in an Internal Medicine hospital setting, which is characterized by frequent one-on-one doctor patient encounters and a case mix that closely resembles that of family practice. We interviewed doctors and patients in the Internal Medicine Department of a (public) teaching hospital and its two satellite hospitals, where doctors mostly see one adult patient at a time and a specific amount of time is allocated to each consultation. These circumstances ensure that the study setting reflects Southeast Asian culture while at the same time it is comparable to settings in which doctor patient communication is studied in Western settings.

## Method

### Participants

We used purposeful sampling to select 20 doctors and 20 patients for the study. Each sub-department of internal medicine at a teaching hospital was represented by two doctors and two patients. The doctors were 10 residents (Doctors 1–10) and 10 specialists (Doctors 11–20). Nine of the doctors were female. Twelve doctors worked at the teaching hospital and four specialists and four residents practised in two affiliated hospitals. We interviewed 20 patients in the internal medicine outpatient clinic of the participating hospitals. Of the participating patients, 10 had an educational background which we defined as low (mostly only elementary education; patients 1–10), and 10 patients had an educational background which we defined as high (mostly bachelor degree; patients 11–20). All participants completed a consent form before the interview.

### Instrument

The first and second authors together conducted in-depth interviews with individual doctors and patients. The interviews with the doctors varied in duration but generally lasted <1 h. The interviews with the patients lasted longer, sometimes up to 2 h. The interviews were audio recorded and transcripts were made within 48 h of the interview.

### Procedure

The grounded theory method was used in order to find out new emerging themes in the context of a paternalistic or one-way doctor–patient communication style in Southeast Asia, by exploring perceptions in relation to literature and cultural characteristics. The specific method used during data collection was in-depth interview (Kennedy and Lingard 2006; Patton 1990). The first and second authors interviewed doctors and patients. The first and second authors discussed the findings of each interview, decided whether additional interview was likely to produce new information, and if so, planned the next interview for the following week. The additional interviews were aimed to deepen exploration on particular cultural issue. This iterative process continued for 26 weeks at which point saturation was reached (Kennedy and Lingard 2006; Patton 1990).

In our previous studies we found a more one-way style of communication in our culture, dominated by the doctors (Claramita et al. 2011a, b). Based on these findings, in this study we interviewed the doctors with questions: “Which factors determine the use of a paternalistic/one-way communication style by Southeast Asian doctors?” Another question was: “Did the doctor use a paternalistic/one-way style intentionally or not?” The word “intentionally” here means that the doctor deliberately used a paternalistic/one-way communication style. If this was the case, it was assumed that the doctor had selected this style from other alternatives although they might initially have been willing to use a more participatory style. We did not put this question directly to the doctors but explored the doctors’ feelings during consultations and how they thought they could improve their consultation style.

The interviews with the patients explored the question: “How do you feel about your doctors’ communication style?” We explored patients’ agreement or disagreement with the doctor’s consultation style, and we observed and explored their non-verbal clues (such as hesitation) in showing their (dis)agreement.

### Analysis

The first and second author coded each transcript independently and met regularly to discuss their findings. The data were explored to find emerging characteristics that could be considered to be typical of Southeast Asian culture and represent factors underlying the use of a one-way communication style.

Based on the results of the interviews, we constructed a framework of cultural and clinical characteristics that can elucidate the use of a paternalistic communication strategy by Southeast Asian doctors and patients.

## Results

### Southeast Asian cultural characteristics

Southeast Asian culture affects doctor–patient communication in several ways. The interviews revealed cultural characteristics that could be placed in two categories: characteristics that reinforce the social distance between doctor and patient and characteristics relating to ‘collectivism’ or closeness of relationships (Table 1).

**Table 1:** Cultural characteristics embedded in the communication style shown by Southeast Asian doctors

Broad categories of characteristics	Cultural characteristics	Doctors’ statements (the unintended hierarchical style)	Statements of patients of low educational level (dissatisfaction)	Statements of patients of high educational level (dissatisfaction)	Interpretations of the quotes and discussion based on the literature
Large gap due to social and cultural hierarchy influenced by	Hierarchical culture	“The principle is: the doctor is the ‘guru’ for the patient!” (Doctor 12: an internist with 2 years’ experience)	“What I feel is actually different to the last time I saw this doctor, but how can I put it? I must obey him, he is the doctor” (Patient 5: low educational level)	“Perhaps the doctors know everything about diseases, but we are the ones who are suffering. They should listen to us. Maybe the doctor will listen to me if I call him mister or madam and not “Doctor” (Patient 12: high educational level)	These quotes reflect the hierarchical social pattern. The doctor is a member of the top level of society and the patients usually come from other levels. The doctors recognize their position in the context of the study. Low education patients feel compelled to obey the doctor because they think the doctor knows best. A high educational level of patient questions the doctor’s judgement, indicating that the patient would feel more comfortable if the doctor was on the same social level
	The importance of maintaining a relationship that is superficially harmonious	“I recognized her as a relative of one of my colleagues. So I thought I could leave it to my colleague to explain the use of the inhaler to her in a more appropriate way. I would not dare to do that. Because I fear it would not be convenient for her to listen to my explanation” (Doctor 20: an internist with 10 years’ experience)	“Well, I hesitated to ask the doctor how to use the medication, because my brother knows him very well. Maybe I can ask somebody else later” (Patient 8: low educational level)	“My husband is a surgeon, he does not necessarily know about using an inhaler. Neither do I. The doctor should have explained it to me!” (Patient 19: high educational level)	These quotes illustrate the doctors’ lack of patient education skills which is exacerbated by the superficial relationship, where politeness and a good atmosphere take precedence over the patient’s concerns. In the past, non-westerners may have been happy with this type of superficial relationship, but we are not sure if this is still the case today. The underlying belief in maintaining harmony is served. The patients are likely to find ways to overcome their dissatisfaction
	The social norm that allows for informal individually modified interpretations	“Communication is an art, like selling *Soto*. The same *Soto* may appear different because it was made by a different seller. You have to choose the one that suits you” (Doctor 14: an internist with 4 years’ experience)	“We often need a doctor who already knows us very well. We do not want to go to great lengths to find a suitable doctor. It is difficult” (Patient 1: low educational level)	“The right doctor should be there every time we need them” (Patient 17: high educational level)	Remarks like “communication is an art” are often made by doctors who have received little communication training. However, in the context of the study, where *Soto* is a special soup that is popular with many people, we interpreted this remark as a reflection of the social norm which accepts informal individually modified interpretations. Doctor 14 concedes that there is a high popular demand for *Soto*. In other words he understands that good communication is important to all patients. However rather than trying to develop a communication style that is effective for everybody, he leaves it up to his patients to choose and allows his students to use any communication style they want This characteristic seems also closely linked to “hierarchical respect”. It could also be interpreted that the doctor was actually trying to say that his Soto or his expertise is superior, so that everybody will turn to him in the end. This situation creates dependency upon senior doctors
	Acceptance of uncertainty	“Well, the patient’s lab test is normal. I think there is nothing wrong with her. I am not sure why she was complaining about such a symptom. I guess she is fine” (Doctor 8: a fourth year resident)	“Although I still feel some discomfort, I suppose I should go home as he told me” (Patient 3: low educational level)	“My lab test is normal for today. But I still feel something troublesome in my chest. Why didn’t the doctor suggest another plan? I will insist to do another series of tests tomorrow” (Patient 12: high educational level)	These quotes are illustrative of a society that is characterized by a low to middle need for certainty. However, the quote from the patient with high education shows that today some patients demand more participation in the care plan
	Lack of awareness of unusual findings or conditions	“The only medicine to deal with his asthma attack is Salbutamol. I cannot prescribe him anything else. Other patients usually do not experience adverse effects from this medicine” (Doctor 7: a fifth year resident)	“… he always gives me the same medicine. But I always experience a tremor …” (Patient 6: low educational level)	“I think this electric connection disturbance in my heart might or might not be associated with the prolonged intake of the hormone pills I took for 9 months during my second pregnancy—whereby I had to deal with a uterine tumour. But why didn’t the internist take my story into consideration?” (Patient 13: high educational level)	Southeast Asian culture places high value on harmonious relations. It is important to avoid conflict. However, efforts to maintain harmony may prevent doctors or patients from paying attention to unusual or unexpected findings. The doctors’ clinical reasoning falls short in identifying the patient’s true problem, while harmony is maintained
The closeness of relationships is influenced by	A strong family support system	“I usually tell hypertensive patients to reduce salt intake in their diet” (Doctor 18: an internist with 8 years’ experience)	“I am willing to do what the doctor told me. But, my wife who usually cooks for me was not there to listen to the doctor … Well my wife runs a small *wrung* (food stall), it will be extra work for her to cook a special menu for me …” (Patient 4: low educational level)	“My sister had already agreed to donate one of her kidneys to somebody who needed it. But then she changed her mind because her husband and children did not permit her to do that and worried about her future condition” (Patient 18: high educational level)	Here we see that patients are surrounded by their close relatives who provide for their everyday needs and who have a strong influence on their clinical decision making. The one-on-one consultation between a doctor and a patient does not seem effective in a Southeast Asian context. The presence of a third party or the closest family member should be facilitated. Especially when the doctor delivers patient education and counselling
	The use of traditional medicine	“Many un-educated people will only obey their traditional healers. If the healer says ‘don’t take these malaria pills, they will not take them” (Doctor 17: an internist with 7 years’ experience)	“Well, the healer lives with us every day. I believe him, he has already cured many diseases among us” (Patient 9: low educational level)	“I don’t believe in those kinds of traditional healers. It is only magic … I already searched the website about my illness. I want to be prepared when I meet the doctor for discussion. But well, sometimes I do not dare to refuse my mother’s suggestion to try an alternative medicine” (Patient 16: high educational level)	These quotes show that the family is a crucial characteristic in the context of this study. A patient never stands alone. Family, neighbours and friends may influence a patient’s decisions including whether to use alternative medicine or not. Traditional medicine is also one of the strong characteristics of the Southeast Asian context These quotes also show that doctors are forced to use a paternalistic style because the conflict between modern and traditional medicine is so strong. It can be very difficult for the doctor to take account of the patient’s perceptions, especially the low educated patients The absence of a tradition of family practice as a broad specialty magnifies this conflict because the doctor and the patient only meet during a brief consultation. The gap between an internist (highly specialized in one area of medicine) and the patient (specialist in his or her own illness) is too wide to engage in context-sensitive patient education and counselling
	The strong non-verbal etiquette of politeness	“Sometimes I do not believe that the patient takes the medicine as prescribed. But how do you check it with the patient? They just say “yes” if we ask whether they take the medicine properly” (Doctor 19: an internist with 9 years’ experience)	“If I say this and that … I do not think it is proper, so I obey the doctor, I do not want any conflict” (Patient 8: low educational level)	“The doctor sometimes tells me about the recent evidence-based medicine. I want to have proof of it, but then I thought about it, and reconsidered, because it may make his job more complicated” (Patient 12: high educational level)	The hesitant behaviour that is exemplified in the quotes was communicated by the patient to the interviewer. We are sure that these hesitant behaviours were communicated as non-verbal politeness in front of the doctor, who did not respond adequately. Such non-verbal behaviour is typical for Southeast Asians who are expected to show respect to others This politeness should not be misinterpreted as acceptance of a superficial relationship and hierarchical respect as mentioned above

Characteristics promoting social distance are: (1) hierarchical respect towards elders or people of higher social status, (2) emphasis on superficial accord rather than on the building of an open, long-term relationship, (3) the social norm that allows for informal individually modified interpretations, (4) a tendency to show hesitant or diffident behaviour, (5) the importance of maintaining harmony and therefore causing unawareness of possible evidence that would challenge ideas. Characteristics relating to the closeness of relationships are: (1) a strong family support system or the communal society, (2) the widespread use of traditional medicine and (3) high value placed on a non-verbal etiquette of politeness.

Doctors unintentionally adhere to behaviour that underlines social distance, while patients seem dissatisfied with that type of behaviour. Patients, by contrast, seem to have developed a tendency to prefer a closer relationship, which doctors unintentionally fail to acknowledge or notice.

### Characteristics of Southeast Asian health care

Three conditions that are inherent to the clinical setting of a Southeast Asian (public) teaching hospital influence the doctor–patient relationship. Firstly, doctors have no role models of the desired doctor–patient communication and students do not actively participate in patient care. Moreover, traditional agrarian behaviour patterns are reflected in long waiting times. These characteristics are illustrated and discussed in Table 2.

**Table 2:** Clinical settings characteristics in a Southeast Asian teaching hospital that influenced doctor–patient communication

Clinical settings characteristics	Doctors’ statements	Low educated patients’ statement	High educated patients’ statement	Literature on specific context
Lack of role model in partnership communication with patient	“This is the model of an old fashioned teacher: what do you feel? Oh okay, this is the medicine.’ A direct and short communication” (Doctor 6: *A third year resident*)	“A good doctor? Doctor A treats me like this … Doctor B treats me like that … Doctor C treats me …” (Patient 9: a low-educated patient)	“That is the role-model, it may mislead the students about proper communication with patients!” (Patient 13: a high-educated patient)	The model described by the resident is a typical model of a traditional doctor from the past. The low-educated patient cannot describe what a good doctor is. The high-educated patient explained that this may be the wrong model to establish a good doctor–patient relationship. From their experiences, both patients have difficulty in describing which example is ideal. Lack of role-model of ideal doctor–patient communication is typical in the context of the study. Role-model is one of the important educational tools
Lack of participation of students in patient-care	“I explained to student A that Mr. X (whom I examined just now) is having chronic obstruction pulmonary dysfunction. Then I ask Mr. X that I think he understand my explanation” (Doctor 3: *a third year resident*)	“The doctor already explained the disease to the young doctor. Well, I do not dare ask anymore questions” (Patient 2: a low educational level)	“That doctor did not talk to me. He talked to the student. When he said okay, I do not think he meant me. I demand further explanation” (Patient 11: a high educational level)	In the context of this study we already knew that students do not participate in patient-care. Students only observe. This clinical education system that is adhered to the unmanaged health care system begun to create confusion for the residents and the patients. No one was sure who is talking to whom. Participation in patient-care is the key for successful clinical education
Traditional agrarian-culture (Doctor 15: an internist with 5 years’ experience)	“I always inspect whether the residents or my students are ready or not in the clinic. Nowadays at 9 o’clock am they are ready. It is good!”	“It is usually like that. I came at 6 o’clock in the morning to queue up. I usually meet the doctor in the afternoon. That is common isn’t it? Well, after waiting for hours, sometimes I forget what to say …” (Patient 7: a low-educational level)	“The clinics open at 8 am. But we are never sure when the doctor will come. We wait for hours but we only meet the doctor for 5 min. What kind of detailed story can be presented then?” (Patient 16: a high-educational level)	One of non-western philosophy is to be calm, they do not targeting anything. This is typical of a traditional agrarian-society when harvest is unpredictable. Doctors still adhere to this kind of traditional agrarian behavior. Amplified by the unmanaged health care-system. The low-educated patient has internalized this unaccepted behavior into something that “common”. The high-educated patient articulates that they do not agree at all. Both patients are unsatisfied

### Dissatisfaction among patients from high and low educational backgrounds

During the interviews, patients expressed dissatisfaction with their doctors’ paternalistic/one-way consultation style. However, they did not articulate this during consultations. If they showed their dissatisfaction at all, it was in an indirect, rather hesitant manner. The behaviour of patients of low educational background during the interviews was best characterized as diffident and hesitant. Exploration of this problem during the interview revealed that the patients were not satisfied with how they acted, but did not know how to overcome their diffidence and express their concerns more openly.

I want to ask questions, but I am afraid the doctor will mind*.*

(Patient 4: a low-educated patient)

Patients with a higher educational level experienced the same sort of hesitation but revealed this only at the end of the interview with the researchers. Their reluctance to explicitly express disagreement or dissatisfaction was not adequately recognized by the doctors.

I can measure the blood pressure myself. Usually it is never this high. But the doctor said that my blood pressure was high. I want to make sure, but to avoid a confrontation; I think I will re-do the examination later.

(Patient 12: a high-educated patient)

### Doctors’ use of a paternalistic communication style is not deliberate

The doctors did not deliberately use a paternalistic consultation style. They said they wished to use a participatory communication style, although the style they actually used was very different. Factors compelling doctors to use a paternalistic style are similar to those we found in a previous study: inadequate communication skills due to no or insufficient training, lack of time due to high patient load, which is inherent in the organization of health care in the hospital, and the doctors’ belief that patients were not prepared for a more participatory style.

I always allow patients to have a discussion with me. But when time is limited, and there are still many many patients waiting outside; I have to end the consultation right away.

(A specialist doctor with 10 years experience)

This quote shows a doctor who is willing to take more time to talk with the patient, but who nevertheless has to end the consultation when he remembers the long queue of patients outside his office. In this particular case, the doctor ended the consultation before discussing the proposed treatment plan with the patient, which could be interpreted as a demonstration of inadequate consultation skills.

## Discussion

Using a partnership doctor patient communication style as generally recommended in Western medicine is no easy task in a culture in which communication is determined by accepted social differences and indirect communication patterns aimed at avoiding conflict and maintaining a pleasant atmosphere. Nevertheless, the interviews in this study revealed that patients, irrespective of educational level, desired a more open communication with their doctor.

A communal society like the Indonesian one is characterized by a strong family support system combined with the use of traditional medicine and a communication style that favours non-verbal expressions of politeness. The main barrier that prevents doctors from adopting a partnership style relates to the social gap between people of perceived lower and higher social levels, with the doctor typically belonging to the latter group. This is a typical phenomenon in a hierarchical culture.

The idea of each of the cultural characteristics may have been found separately in many previous studies (Galanti 2008; Hofstede 2003; Raelin 2000; Iragiliati 2006; Geertz 1976). However, strength of this study is that those cultural characteristics are related to doctor–patient communication, in view of the prevailing one-way communication style. The findings can be use accordingly to construct a communication skills model that suited the Southeast Asian context. In the long run, the model can be used for training health professionals in these cultures.

A possible communication approach that Southeast Asian doctors might use to modify their communication style in the desired direction might be found in the culturally accepted close relationship in the community and mutual support between family members. Doctors could try to mirror that relationship during consultations thereby harnessing its positive effects to the delivery of optimal health care in a way that is congenial to Southeast Asian doctors’ and patients’ expectations.

Some of the characteristics of consultations that we found in this study resemble what happens in Western settings. “Uncertainty” about the treatment plan and failure to recognize an atypical condition of a patient or ‘representativeness error’ are common in both settings of Western and non-Western (Groopman 2007).

Your thinking is guided by your prototype, so you fail to consider possibilities that contradict the prototype…

(Groopman 2007, page 44)

However, Western doctors may be more inclined to reflect on such errors and share their reflections with the more junior doctors. At this point, Western doctors tend to communicate their thinking with junior doctors; in an equal manner, in order to support the juniors’ learning process. Whereas, in the context of this study, we found no indications that doctors reflect on their actions. If we had encountered reflection during the interviews, it would likely have intensified the hierarchical pattern, which is legitimized by seniority and widens the gap between doctors and the young doctors. At the end, this phenomenon widens the gap between doctors and patients.

Recently, Indonesian medical schools’ curricula have become more oriented towards competence-based education (IMC 2006). Seven competencies have been indentified, including socio-behavioural competences in which communication and professionalism are two of the competences. However, our study revealed that the implemented curriculum was different from the desired curriculum, which mostly emphasised biomedical sciences (Claramita et al. 2011c). It seemed that medical teachers in the context of the study had difficulty in teaching topics that considered patients’ concern.

Conflict between a more ‘modern’ or open-minded partnership style communication and the ‘traditional’ one-way style of communication views is a familiar phenomenon in both Western and Southeast Asian cultures. The way people stick to culturally determined patterns of behaviour (although not on purpose) and ignore other options is associated with their interests (Zeichner 1988). Adherence to a paternalistic/one-way communication style seems to fit a cultural pattern in which doctors’ interests take priority over patients’ interests.

The clinical education context in Southeast Asian teaching hospitals should also be considered as an important factor with regard to doctor–patient communication. Currently, medical clerkship students only observe and do not actively participate in patient care. If “participation” in any clinical education context does not occur, medical graduates may still not fully understand how to invite patients’ participation in the consultation. The agenda of people perceived from the higher hierarchical level (e.g. medical teachers and medical doctors) takes precedence over the agenda of people perceived from the lower hierarchical level (in this case: medical students and patients).

The way outpatient clinics are organized strongly impacts the way doctors manage their time and work. Patients do not make an appointment but turn up at the clinic and wait their turn. This means that doctors do not know in advance how many and which patients they will see, and patients may see a different doctor each time they come to the clinic. Also doctors usually work in different hospitals and practices. In the absence of a well-planned clinic schedule and the daily rush of work forces doctors to approach consultations as a routine task driven by high production targets in terms of the number of patients seen. There is little room for them to treat patients as individual guests with specific needs. The same lack of structure may explain why the doctors we interviewed had little opportunity to reflect on how they might improve clinical education. This compares unfavourably to Western doctors who may work in one clinical setting for decades (Groopman 2007; Dornan 2006; Dolman et al. 2008).

In this study, all patients, irrespective of educational background, expressed a preference for a partnership consultation style. Studies in Western contexts have shown that the doctor–patient relationship becomes closer as patients become more knowledgeable about their illnesses and participate more actively in the consultation (Lings et al. 2003; Murray et al. 2007). In our study, the less educated patients indicated that it was difficult for them to invite their doctor to use a partnership communication style. However, this does not mean that they did not wish or try to do so. Less educated patients could be helped to modify their communication style by encouraging them to express their concerns to the doctor (Fiscella et al. 2000).

The doctor holds the key to the participatory consultation. However, doctors are also facing a formidable barrier of high patient load due to inefficiencies in the health care system. Moreover, even if more time were available for a consultation (for example during a house call or ward visit) a doctor who is unskilled in communicating with patients is unlikely to be able to engage in participatory doctor–patient communication.

We recommend further investigations on a suitable communication skills model, tailored to the needs of Southeast Asian patients and doctors who seem critical of this point. Such a model could pave the way towards the preferred style of doctor–patient communication in a Southeast Asian context. It could incorporate aspects of communication skills that are characteristic of the collectivistic culture, such as context sensitive individual and community health education skills (Werner and Bower 1982) including the use of traditional medicine in harmony with modern medicine (Geertz 1976; Galanti 2008). When doctors in the context of this study have better skills of exploration and facilitation during consultations, they are better able to encourage their patients to express their worries and concerns more openly. Furthermore, it is important to be aware that “yes” in a Southeast Asian context may not be a true “yes” but simply an automatic, socially correct, polite and respectful response (Raelin 2000).

For other settings we recommend further intercultural communication study, to complement the communication skills model that usually originates from the Western world, with local evidence (von Fragstein et al. 2008). Environment, culture and illness experiences are strong factors for every patient which should be taken into account during doctor–patient consultations (Helman 1994).

One limitation of this study is that the doctors’ views were not explored exhaustively. The interviews lasted less than an hour, because doctors are always under pressure to attend to more patients or meetings. Appointments for interviews that we made with doctors had to be rescheduled many times, due to the multiple roles doctors undertake in the health care system. Nevertheless, we continued to collect data until saturation of information was reached. Another limitation of this study is that the interviews were conducted in an outpatient setting only. Further investigations should include patients’ and doctors’ experiences in hospital wards. The exclusive participation of Javanese doctors and patients may also be a limitation. It remains to be investigated to what extent they are truly representative of the broader Southeast Asian culture.

So far we have uncritically used the Western communication skills model in a Non-Western setting. Our increased insight may provide guidelines to create a more appropriate Southeast Asian model for doctor–patient communication. Time for change!
